# Variability Between Clinical Pharmacogenetics Implementation Consortium (CPIC^®^) Guidelines and a Commercial Pharmacogenetics Laboratory in Genotype to Phenotype Interpretations For Patients Utilizing Psychotropics

**DOI:** 10.3389/fphar.2022.939313

**Published:** 2022-06-24

**Authors:** Christopher Blazy, Vicki Ellingrod, Kristen Ward

**Affiliations:** ^1^ Department of Clinical Pharmacy, College of Pharmacy, University of Michigan, Ann Arbor, MI, United States; ^2^ Clinical Pharmacy Department, Michigan Medicine, Ann Arbor, MI, United States

**Keywords:** cytochrome P-450 CYP2D6, cytochrome P-450 CYP2C19, pharmacogenomics, star allele, phenotype, genotype, psychotropics

## Abstract

Clinical practice environments without in-house pharmacogenetic testing often rely on commercial laboratories, especially in the setting of pharmacogenetic testing intended to guide psychotropic use. There are occasionally differences in phenotype assignment and medication recommendations between commercial laboratories and the Clinical Pharmacogenetics Implementation Consortium (CPIC). This may be problematic as many institutions that implement pharmacogenetics consider CPIC to be an important source of guidelines for recommended prescribing actions based on genetics, as well as a tool towards standardizing pharmacogenetics implementation. Here, we completed a retrospective chart review of our academic health system’s (Michigan Medicine) electronic health record with the goal of comparing phenotypic assignment of *CYP2D6* and *CYP2C19* genotypes between the commercial pharmacogenetic lab used most at our institution, and CPIC. Ultimately, we identified 205 patients with available pharmacogenetic results from this lab. The prevalence of conflicting phenotype assignment was 28.8% for *CYP2D6* and 32.2% for *CYP2C19* genotypes when comparing the commercial lab to CPIC guidelines. In several cases, the phenotypic assignment differences for antidepressants led to significant differences in medication recommendations when comparing the commercial lab report and CPIC guidelines. These results may also have implications for medications outside of psychiatry with recommendations for dose adjustments based on CYP2D6 or CYP2C19 metabolizing phenotype.

## Introduction

Major depressive disorder (MDD) is one of the leading causes of morbidity and disability in the United States (James et al., 2018). Reviews of international data also demonstrate that while rates of deaths due to all diseases and disability-adjusted life years (DALYs) appear to be decreasing over time, these rates specific to mental health have been increasing, and an approximate 19% of years lived with disability were due to mental health and addictive disorders (Rehm and Shield, 2019). Although pharmacological management of MDD is a key component of treatment for most patients with moderate-to-severe depression, rates of remission (complete resolution of symptoms), response (50% reduction in symptoms), and no response can be roughly described by the rule of thirds: 1/3 remit, 1/3 respond, 1/3 do not respond (Rush et al., 2006; Sinyor, Schaffer and Levitt, 2010). Currently, there are no diagnostic tests available to select the precise medication that will be efficacious for a patient in treating their MDD. There are pharmacogenetic variants that have been associated with significant changes in drug metabolism, and guidelines have been developed to provide recommendations for select psychotropic medications when considering genetically predicted metabolizing ability.

The Clinical Pharmacogenetics Implementation Consortium (CPIC) is one organization dedicated to the creation of pharmacogenetic guidelines (Caudle et al., 2014; Relling et al., 2020). They are composed of an international group of researchers, patient advocacy groups, and clinicians with expertise in pharmacogenetics. As of April 2022, CPIC has published 26 guidelines (CPIC Guidelines List, 2022) with recommendations for specific gene-drug, or gene-drug class interactions, and they have been utilized by many early adopters of pharmacogenetics in the United States (Hicks et al., 2016; Ja et al., 2017; Volpi et al., 2018). Relevant to psychiatry, they have published guidelines for selective serotonin reuptake inhibitors (SSRIs) (Hicks et al., 2015), tricyclic antidepressants (TCAs) (Hicks et al., 2017), and carbamazepine and oxcarbazepine (Phillips et al., 2018).

The providers at our academic health system (Michigan Medicine, Ann Arbor, MI) who order pharmacogenetic tests most commonly use a commercial pharmacogenetics laboratory. Starting in 2018, our pharmacy team began reviewing pharmacogenetic results as part of a newly implemented consultation group. The foundations of most of our medication recommendations are CPIC guidelines and Food and Drug Administration approved drug labels, while also considering important clinical considerations like phenoconversion, drug-drug interactions, organ function, and past medication response. Through these consults, it became apparent that there were several areas of genotype to phenotype translation disagreement between the commercial lab and CPIC. These discrepancies were most readily apparent for *CYP2D6* and *CYP2C19*, which are also the genes on the commercial report with the largest number of associated CPIC guidelines for psychotropic medications. Here, we present a descriptive analysis of discrepancies between CPIC and the commercial lab for *CYP2D6* and *CYP2C19* genotype to phenotype translations discuss how these discrepancies may ultimately impact medication recommendations.

## Methods

A retrospective chart review of patients with pharmacogenetic testing results, ordered to guide psychotropic use, was performed. Patients were identified through the Electronic Medical Record Search Engine (EMRSE) (Hanauer et al., 2015) with the key phrase “pharmacogenetics pharmacist interpretation of results” from January 1, 2018 to June 1, 2021. EMRSE is a tool capable of searching text within the EHR, and this phrase was chosen because it is unique to our note template containing pharmacist recommendations after PGx results review. Inclusion criteria required patients to have completed a commercial pharmacogenomic test from the dominant commercial product at our institution (GeneSight Psychotropic panel), and that the results were added to their electronic health record (EHR). Patients of all genders, ethnicities, ages, health conditions, and reason for testing were included. Patient charts were reviewed for pharmacogenetic testing results for *CYP2D6* and *CYP2C19*, and the commercial lab-reported genotype and phenotype results for each patient were recorded. Additionally, phenotypes were assigned for each patient based on CPIC guidelines for *CYP2D6* and *CYP2C19* based on the genotyping results of the commercial lab.

A descriptive comparison between the commercial pharmacogenomic phenotype test results and CPIC guideline phenotype results were completed for CYP2D6 and CYP2C19. This study was approved by our Institutional Review Board (IRB).

## Results

A total of 207 patients were identified who had psychotropic pharmacogenetic testing results in their electronic health record, in addition to having pharmacist involvement in the result review. Two patients were removed due to pharmacogenetic testing that was not done by our commercial lab of interest, leaving 205 patients. Out of 205 patients, discrepancies between the commercial lab phenotype designation and CPIC phenotype, based on the reported genotype for *CYP2D6* were found in 59 (28.8%) patients. Of the 205 patients reviewed, discrepancies between the commercial lab phenotype designation for *CYP2C19* and CPIC phenotype were found in 66 (32.2%) of patients. Table 1 details the most common sources of disagreement in genotype-to-phenotype translation between the commercial lab and CPIC. In total, CYP2D6 phenotype disagreements were identified for 15 unique genotypes, and only 3 for CYP2C19.

**TABLE 1:** Common sources of *CYP2D6* and *CYP2C19* genotype-to-phenotype interpretation disagreement between the commercial laboratory and CPIC.

Genotype	Frequency N (%)	CL Phenotype	CPIC Phenotype
***CYP2D6^a^***			
*2A/*2A	15 (7.32)	Ultrarapid	Normal
*2A/*4	12 (5.85)	Normal	Intermediate
*4/*41	9 (4.39)	Poor	Intermediate
***CYP2C19^a^***			
*1/*17	50 (24.39)	Normal	Rapid
*2/*17	15 (7.32)	Normal	Intermediate

*CYP2D6* and *CYP2C19* genotype, frequency found in patient population, commercial laboratory phenotypic interpretation, and CPIC, phenotypic interpretation. CL: commercial laboratory.

a Data provided for individual genotypes when they occurred in at least 4% of patients (Hicks et al., 2015; Hicks et al., 2017).

## Discussion

The results of our retrospective chart review demonstrate that considerable variability exists in the commercial lab designation of phenotypes for CYP2D6 and CYP2C19 as compared to CPIC phenotypic designations of the same genotype in our patient population. Conflicting assignments of phenotype for *CYP2D6* and *CYP2C19* genotypes were 28.8 and 32.2%, respectively.

To get a sense of the potential implications of these results, select antidepressants with *CYP2D6*-and *CYP2C19*-based CPIC guidelines are highlighted in Table 2. The final column provides how CPIC recommendations would differ for the discrepant phenotypes. For example, with respect to SSRIs, the difference between an intermediate and normal metabolizer phenotype of a given genotype would not lead to differential medication recommendations. In contrast, with respect to tricyclic antidepressants (TCAs), patients with a CYP2D6 normal metabolizer phenotype would be recommended to start standard initial dosing of TCAs, but for the ultrarapid metabolizing phenotype, the recommendation would be to avoid TCAs altogether (or to consider use with therapeutic drug monitoring).

**TABLE 2:** Example discrepancies in antidepressant recommendations based on phenotype.

Example Medication	Genotype	CL Phenotype	CPIC Phenotype	Potential discrepancy in CPIC recommendations for different phenotypes
***CYP2D6***				
Nortriptyline (any TCA)	*2A/*2A	Ultrarapid	Normal	*Ultrarapid*: Consider alternative
				*Normal*: Standard starting dose
Nortriptyline (any TCA)	*2A/*4	Normal	Intermediate	*Normal*: Standard starting dose
				*Intermediate*: 25% reduction in starting dose
Nortriptyline (any TCA)	*4/*41	Poor	Intermediate	*Poor*: Consider alternative *Intermediate*: 25% reduction in starting dose
***CYP2C19***				
Escitalopram or citalopram	*1/*17	Normal	Rapid	*Normal*: Standard starting dose
				*Rapid*: Consider alternative
Escitalopram, citalopram, or sertraline	*2/*17	Normal	Intermediate	*Normal*: Standard starting dose
				*Intermediate*: Standard starting dose

Table 2: Highlighted discrepancies in medication recommendations based on different phenotypic interpretations of *CYP2D6* and *CYP2C19*. TCA: tricyclic antidepressant (Hicks et al., 2015; Hicks et al., 2017).

During the time period of pharmacogenetic testing results reviewed here, the CPIC guidelines for SSRIs and TCAs were updated in a standardization project with CPIC and the Dutch Pharmacogenetics Working Group (DPWG) (Caudle et al., 2020, p. 2). As part of this project, the activity score for the *10 allele for *CYP2D6* was downgraded in activity from 0.5 to 0.25. This update did not significantly impact our descriptive analysis here, as identified discrepancies were maintained before and after the change in *CYP2D6* *10 activity score. For example, per the commercial lab a *CYP2D6* *10/*10 carrier was classified as a poor metabolizer, but for CPIC this genotype would be a normal metabolizer (pre-2019 adjustment) or intermediate metabolizer (post 2019-adjustment).

It is important to note that some extent of discrepancy in genotype-to-phenotype interpretation would be expected, based on previous research. A study by Bousman et al. demonstrated this clearly when they noted a multitude of discrepancies when reports from four pharmacogenetics labs with psychotropic panel products were compared for the same 5 patients (Bousman and Dunlop, 2018). Besides stressing the fact that tests and decision support tools vary lab-to-lab when compared for the same set of patients, it also raises the questions as to how congruent pharmacogenetic tests are with expert guidelines.

Additionally, a post-hoc analysis of the randomized controlled trial investigating the potential impact of GeneSight-guided vs. treatment-as-usual prescribing on outcomes in patients with depression (GUIDED) (Greden et al., 2019) looked at a subset of patients (191) with an antidepressant drug level (Rothschild et al., 2021). They compared the ability of their combinatorial gene approach (which includes *CYP2D6*, *CYP2C19*, and *CYP3A4* genetic variants) to CPIC guidelines to predict serum concentrations of escitalopram or citalopram. The authors concluded that their proprietary algorithm was superior to single gene approaches utilizing CPIC guideline-assigned phenotypes to predict serum concentrations. Interpretation of the results are complicated by not knowing the timing of the last dose in relationship to the blood draw. Given the proprietary algorithm used by the company to make medication recommendations, it would also be difficult to compare medication recommendations for GeneSight vs. CPIC-assigned phenotypes directly, although this study addressed the commercial lab-CPIC discrepancies in phenotype assignment which ultimately influences medication recommendations.

What we would like to stress with the results from the comparison made here, is to encourage clinicians to review the genotype data and to take that into consideration when making recommendations for their patients. With respect to psychotropics, and particularly speaking towards the relatively common *CYP2C19* *1/*17 variant, in our practice we do draw attention to this phenotype discrepancy between the commercial lab and CPIC. One of the reasons we do this is because many of our patients have failed a trial of at least escitalopram or citalopram prior to a pharmacogenetic test being ordered. This can be an opportunity to discuss dosing, or to potentially consider a trial of a medication not primarily metabolized by CYP2C19, if appropriate. Another important consideration that lends itself to looking at the actual genotype and not just the predicted phenotype is for patients with polypharmacy who may be taking non-psychotropic medications with CPIC guidelines (or will take them in the future). Examples of non-psychotropics with *CYP2C19* or *CYP2D6* guidelines include clopidogrel (Lee et al., 2022), tamoxifen (Goetz et al., 2018), and proton pump inhibitors (Lima et al., 2021).

Finally, it is also important to note limitations of our study: this is a brief research report describing discrepancies in phenotype assignment, but it does not provide detail on how these discrepancies may (or may not) impact clinical outcomes with respect to psychotropic and non-psychotropic medication management. A study appropriately powered to identify potential statistically significant findings would need to be large when considering results from the randomized controlled trial with almost 1,400 patients utilizing the GeneSight product which demonstrated that nearly 80% of patients were already taking a medication congruent with their GeneSight reports at baseline (Greden et al., 2019). Ideally, in the future the discrepancies reported here won’t be as much of an issue as discrete result storage for pharmacogenetic variants becomes more widely available. With discrete storage of pharmacogenetic results, institutions can utilize clinical decision support tools to help providers sort through the potential implications of variance between our guideline organizations and commercial laboratories in how test results are presented and interpreted (Gammal et al., 2021).

## Conclusion

In review of patients in our health system who were ordered panel pharmacogenetic testing from a commercial lab to guide psychotropic use, we identified conflicting assignments for 28.8% of *CYP2D6* genotypes and 32.2% for *CYP2C19* genotypes when comparing CPIC and commercial lab genotype-to-phenotype translations. Depending on the specific discrepancy, the discordant phenotype assignments may be associated with different medication recommendations when considering CPIC guidelines. It is unclear how much potential impact the results of this study may have on patient care, but the results are shared here to draw attention to the importance of independently evaluating commercial lab genotypes when interpreting the reports for all medications patients may be taking with relevant CPIC guidelines.

## Data Availability

The original contributions presented in the study are included in the article/Supplementary Materials, requests for the full dataset can be submitted to the corresponding author.
